# Fat Content Effect in the Measurement of T2* for Iron Quantification in the Liver in Patients With Suspected Myocardial Iron Overload

**DOI:** 10.1186/1532-429X-18-S1-P285

**Published:** 2016-01-27

**Authors:** Flavia P Junqueira, Juliano L Fernandes, Gustavo A Pinto, Sandra R Loggetto, Maria Cristina Purini

**Affiliations:** 1 Cardiovascular Imaging, Jose Michel Kalaf Research Institute, Campinas, Brazil; 2 DASA, Clinics Delboni Auriemo and Lavoisier, Sao Paulo, Brazil; 3 Centro de Hematologia de Sao Paulo, Sao Paulo, Brazil

## Background

Iron quantification using T2* has become a routine non-invasive procedure for assessment of myocardium and liver in patients with regular transfusions. Cardiologists and cardiovascular radiologists are progressively measuring iron overload in the liver associated with myocardial overload. It is currently unknown if potential interference of high fat content in the liver may result in discrepant iron estimations. We sought to evaluate whether the use of regular T2* sequences would be confounded by fat in comparison to a commercial software with fat-water separation.

## Methods

Twenty-six patients with clinical indication for liver and myocardial iron assessment were studied using a 1.5T scanner (GE Optima 450). In all patients, an axial image of the liver was acquired using a multi-echo single breath-hold GRE sequence with 10 TEs and fat saturation followed by an image using a three-dimensional volumetric imaging sequence (IDEAL-IQ, GE Healthcare) with six echo times. For the T2* GRE sequence, a region of interest (ROI) was drawn through the right lobe of the liver and the mean signal intensity for each echo time used to fit the T2* decay curve with the formula SI = Ke^-TE/T2*^ with nonlinear fit and truncation to correct for high iron levels. For the commercial sequence, automatic R2* and fat-fraction maps were created automatically by the software and a ROI was traced on these maps to obtain R2* (1/T2*) and fat fraction (FF) percentage values. Patients were divided according to FF (abnormal >10%) and liver iron concentration (abnormal if T2* <15.4 ms). A comparison of T2* values using both softwares was performed and analyzed according to fat and iron concentrations.

## Results

**Figure 1:**
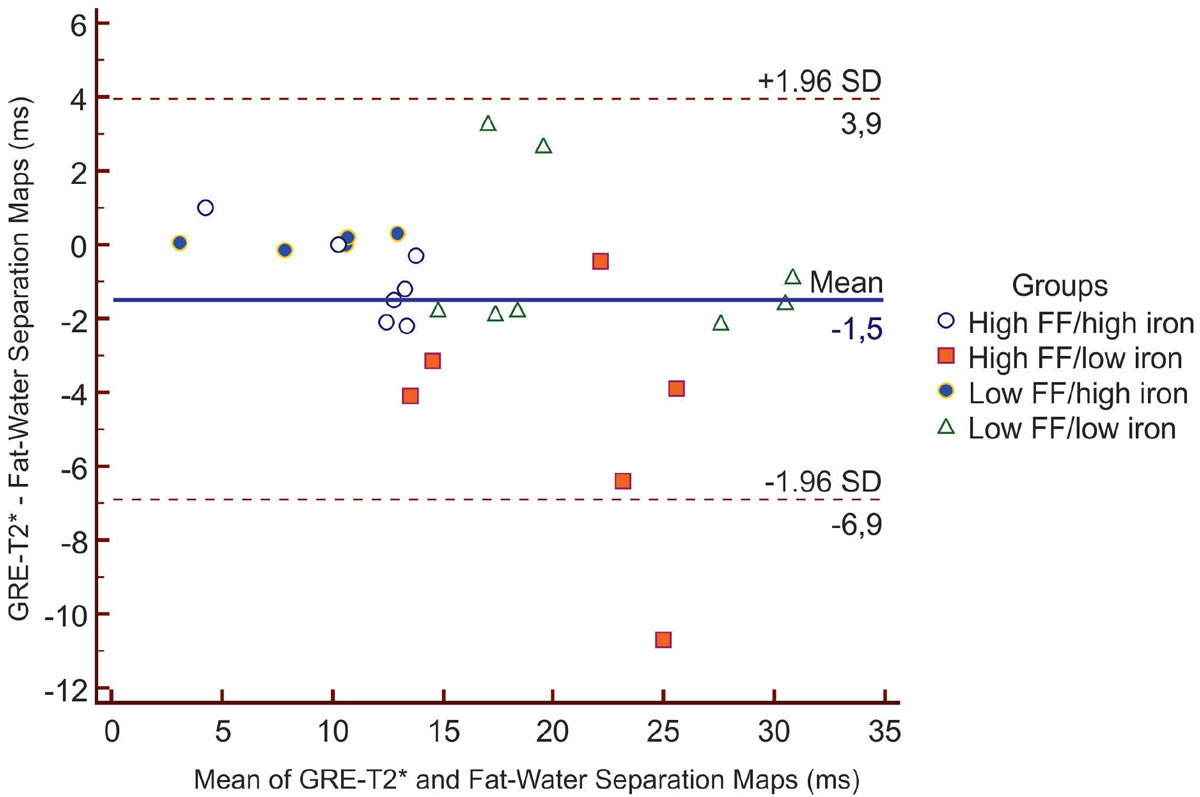
Figure 1

The mean age of participants was 44 ± 14.2 years with 73% males. The fat-water separated results yielded a mean T2* of 17.1 ± 8.0 ms versus GRE-T2* of 15.6 ± 7.0 ms (P=0.01) - Figure 1 shows a Bland-Altman plot of the differences in T2* using the GRE-T2* sequence versus fat-water separation maps. Each subject was classified according to the initial content of iron and FF into low/high groups.. In the group with high levels of liver iron (n=12), this difference was not significant with a mean difference of -0.49 ms (95% limits -2.5 to 1.5 ms). Significant discrepancies in T2* values were only observed in patients with low liver iron content (n=14) with differences of -2.3 ms (95% limits -9.1 to 4.5 ms, P=0.03). The overall median FF was 9.5% (IQR 4.0 to 16.0%) with significant differences in T2* measurements observed only in the group with high FF (17.0 ± 7.5 vs 14.4 ± 5.3 ms, P=0.01). In the group with normal FF (n=13), no differences in T2* were found (17.1 ± 8.8 vs 16.9 ± 8.3 ms, P=0.59).

## Conclusions

With traditional GRE-T2* sequences, fat content of the liver will not significantly affect usual T2* measurements in patient with either low fat content (FF < 10%) or abnormal iron stores (T2* < 15.4 ms). In patients with normal liver iron concentrations and high fat content, GRE-T2* underestimates the liver T2* values.

